# Early parent-child intervention with Dialogic Book-Sharing: effects on child communicative and socio-emotional development and on parenting. Study protocol for a multicentre randomised controlled trial in Italy

**DOI:** 10.1186/s13063-024-08232-4

**Published:** 2024-06-19

**Authors:** Cena Loredana, Trainini Alice, Murray Lynne, Cooper Peter, Calza Stefano, Belluardo Mauro

**Affiliations:** 1 Department of Clinical and Experimental Sciences, Section of Neuroscience, Observatory of Perinatal Clinical Psychology, University of Brescia, Viale Europa 11, Brescia, 25123 Italy; 2 School of Psychology and Clinical Language Sciences, University of Reading, Reading, UK; 3 Unit of Biostatistics and Bioinformatics, Department of Molecular and Translational Medicine, University of Brescia, Brescia, Italy; 4 Unit of Neuroscience, Department of Medicine and Surgery, University of Parma, Parma, Italy

**Keywords:** Early childhood development, Parenting intervention, Book-sharing, Italy

## Abstract

**Background:**

Research in the neurosciences has highlighted the importance of intersubjective relationships in promoting neuromental development of the child. Children’s learning in early childhood occurs mainly in a dyadic context of an interaction with their parents: from this perspective, good dialogic parent–child communication is required to be promoted also through good educational practices. Dialogic Book-Sharing (DBS), a dialogic form of parent–child communication through the use of wordless picture books, provides a privileged ‘intersubjective’ space and is highly effective in promoting communication, language, attention, behavioural development and the parent–child relationship. DBS programme, successfully previously trialled in South Africa and the UK, will be applied for the first time in Italy for research purposes in Italian health, educational and maternal-child centres.

**Methods:**

A multicentre randomised controlled trial is being conducted to evaluate DBS parenting intervention for children aged between 14 and 20 months. Parent–child dyads are randomly allocated to a book-sharing intervention group or to a wait-list control group. In the intervention, parents are trained in supportive book-sharing with their children by local staff of the centres. DBS intervention is carried out in small groups over a period of 4 weeks. Data are collected at baseline, post-intervention and at 6 months post-intervention with a questionnaire and video recording of parent–child interaction.

**Discussion:**

DBS programme in early childhood could enhance the educational resources offered by Italian health, educational and maternal-child centres, in support of child’s development and parenting. DBS represents a strategic opportunity for bringing about positive effects, also in terms of prevention of socio-emotional and cognitive difficulties. As such it represents a promising response to the new social, health and educational needs of the post-COVID-19 pandemic era caused by the social isolation measures. Furthermore, the application of the DBS methodology is a way to promote the use of books, and thereby counteract the excessive use of technological devices already present in early childhood.

**Trial registration:**

The trial is registered on the International Standard Randomised Controlled Trial Number database, registration number ISRCTN11755019 Registered on 2 November 2023. This is version 1 of the protocol for the trial.

**Supplementary Information:**

The online version contains supplementary material available at 10.1186/s13063-024-08232-4.

## Introduction

### Background

In the first years of life, non-verbal communication and language skills develop rapidly, and children learn that their caregivers’ behaviour contains important information about the world [1, 2]. One of the first aspects of early parent–child interactions that help child development is the emergence of joint visual attention between an adult and an infant about a common focus, for example, an object. Another critical aspect of early development that underpins shared understanding and communication is the acquisition of language [3]. Language is a skill that influences other domains, such as social and cognitive development, and requires social interaction [4–6]: language learning relies on children’s desire to imitate [7] and on their appreciation of their caregivers’ communicative intentions [8]. Young children’s learning occurs mainly in a dyadic context of an interaction with a knowledgeable caregiver: from this perspective, good dialogic parent–child communication that involves reading to the child at an early age is highly effective in promoting communication and language development [9–11]. Research on parent–child shared-reading interventions in early childhood [12] has highlighted the benefits for early language acquisition [13] and for accelerating language development [14]. Benefits for child’s linguistic development occur following regular and dialogic reading [15], results confirmed by meta-analyses [16, 17]. Language learning through situation-specific practices, such as reading, in which parents label items more frequently than during other activities such as playtime or mealtime, provides children with particularly consistent and informative linguistic feedback [18, 19]. By reading books to children, caregivers support a wide range of skills. These include reinforcing the acquisition of new words and concepts [20, 21], promoting the child’s literacy skills [22] and providing a ‘lexical reservoir’ that widens vocabulary [23]. In addition, the activity of parent–child shared reading has a significant impact on the development of preschoolers’ abstract language [24], syntactic quality, complexity of sentence construction [25], literal and inferential language [26], listening comprehension, phonemic awareness [27] and receptive and expressive vocabulary [28].

The enrichment of the child’s expressive vocabulary in early childhood is promoted by the cognitive ability to make predictions, an ability that is enhanced by shared reading with the adult: preschoolers develop their language skills when they have opportunities to predict upcoming information during shared reading, through strategic pauses or targeted questions [29]. Shared-reading also increases preschoolers’ oral narrative skills [30]. Improvements in narrative comprehension have shown that conversational reading programmes can also support broader language skills, such as inferential understanding [31], where the opportunity that adult–child book sharing presents for initiating conversations may be key to its effectiveness [32], and where parent-infant conversations promote the parent’s use of metacognitive language [33]. The adult and the child can focus on common aspects of interest in the book by looking at the illustrations, asking questions and talking about the story [34]. Furthermore, parent–child book-reading is a favoured context for parents to prompt mental state discussion with their children [35–37], important for the development of theory of mind skills [38, 39]. Indeed, it has been found that such parental behaviour during picture book reading is associated with child social understanding [40, 41], with positive effects particularly evident in peer relationships [38, 42, 43] and prosocial behaviour [44]. These skills are important as they are associated with subsequent school progress and literacy [45, 46] and predict later educational progress [47].

A structured parent–child shared reading intervention, different from simply reading a book by an adult to a passively listening child, which includes the aforementioned aspects, was developed in the ‘Dialogic Book-Sharing’ programme (DBS) [48]. This methodology consists of a dialogic form of parent–child communication through the use of wordless picture books, beneficial for children in early childhood and preschool age. The colourful illustrations that carry the story line in these books attract the attention of children who do not yet know written language. However, the narrative and textual content of any book does not define a good DBS intervention, but rather it is the interaction that is activated between adult and child which stimulates, through a structured dialogue, lexical learning, attention, and, above all, the awareness and verbalisation of emotions, thoughts and relationships [49]. The main objective of DBS is to share with the child what the images narrate, respecting the pace and the rhythm of the child; thus, a circularity and a reciprocal exchange of parent–child ideas and emotions are facilitated. The DBS training methodology consists of progressive sessions, in a group learning context, in which trained facilitators present ways of using the book to parents, according to a defined programme, where parents learn the methodology and then apply it autonomously in daily life with their children [49]. This methodology requires that the adult pays attention to what interests the child, following their interest, and actively involves them by naming and indicating what the child is looking at, formulating comments, asking questions and sustaining their interest through the use of vocalisations and gestures [48, 50]. Specific conversational techniques used by the adult are included, such as asking questions aligned with the child’s ‘proximal development zone’ [51]. The content of the book is linked to the child’s experience, a technique widely adopted in book-sharing interactions [52, 53] that promotes child language development [54, 55]. DBS methodology thereby allows the child to develop linguistic and cognitive skills and is of potential benefit to children’s future ability to adapt to the school environment [56].

Several randomised controlled trials have been conducted to evaluate the effectiveness of the DBS methodology, especially in populations where, by virtue of exposure to socioeconomic and familial risk factors, children have poor language [57], cognitive [58] and literacy skills [59]. In these contexts, the sharing of books has been found to be particularly beneficial for narrowing the social gap. In a pilot randomised controlled trial (RCT) in South Africa, socially disadvantaged mothers’ behaviour while sharing books or toys with their 14–18-month-old children was assessed before and after a DBS intervention programme [48]. Positive outcomes were found for child attention and language: compared to a play-support control group, mothers receiving DBS training became more sensitive, facilitating and elaborative with their infants, and the infants evidenced a significantly greater increase in expressive and receptive language, as well as in sustained attention. A follow on full RCT study conducted in the same South African community with carer-infant dyads of 14–16-month-olds found the same benefits for child language and attention [60], as well as benefits to prosocial behaviour social awareness [49]. The benefits to child language and attention were shown to have been mediated by improvements in maternal sensitivity and reciprocity. A recent review and meta-analysis of 19 RCTs of DBS also confirmed the efficacy of DBS interventions for improving the development of child language [17]. Notably, Dowdall et al. reported that dialogic book-sharing is effective even when caregivers had low levels of education, underlining the importance of including this methodology in programmes that aim to support early literacy and language development in infants and children, especially in socially disadvantaged contexts. The benefits of Dialogic Book-Sharing have also been studied in European populations: for example, a study conducted in UK on dyads including carers of 28- and 45-month-old children [56] found substantial benefits especially concerning carers’ sensitivity and cognitive scaffolding [61].

On the basis of extensive research, it has been argued that Dialogic Book-Sharing provides a privileged ‘intersubjective’ space for the promotion of child learning and cognitive and language development [50]. The intersubjective process that arises in DBS interactions provides a contained space for joint visual attention, in a physically close intimate setting, that is associated with shared physiological and affectively positive states [62]. The affective messages that the caregiver transmits allow the relational experience to be memorised [63–65] and can contribute to the promotion of secure attachment [25, 66, 67]. Current research in the neurosciences has highlighted the importance of intersubjective relationships as well as the neurobiological underpinnings of such processes [68]. Within the intersubjective space of the parent–child reading relationship, one aspect that we believe to be of fundamental importance is the ‘affective predispositions’ with which parents conduct book-sharing ‘with’ their children [69]. These dispositions are mainly transmitted through non-verbal communication. Visual-facial, tactile-gestural, auditory-prosodic expressions (the prosody of the mother’s voice is already memorised and learned during the prenatal period) [70] constitute the primary mother-infant affective communications, including empathic mirroring during mutual gaze transactions. From a neurobiological perspective, one of the most supported models posits the existence of a distributed network involved in bodily and non-verbal interactions, especially for facial expressions [71]. A crucial component of such model is represented by the Mirror Neuron System (MNs), with sensorimotor neuronal networks that are active during both the production and the passive observation of actions and of emotional facial expressions [72–75]. In addition to premotor and somatosensory parietal regions, such network includes also specific limbic structures (i.e. the anterior insula, the amygdala and the anterior cingulate cortex) which are also involved in the modulation of autonomic and vegetative responses coupled with expressions of emotions [74–77]. Several studies in typical developing individuals as well as in pathological conditions (such as congenital facial palsy) [78] suggest that such network could crucially subserve critical bodily and non-verbal aspects of intersubjective processes since early social interactions [68, 79, 80], such as reciprocal behavioural synchronicity [81–83], intentions and emotion processing [84] and reciprocal ‘affective attunement’ [68, 78, 84–86].

Indeed, if the mother is psychobiologically attuned to her infant, she synchronises the spatiotemporal behaviour pattern of her stimulation with the spontaneous manifestations of the child’s organic rhythms and promotes their emotional regulation [87]. The mother accepts the non-verbal expressions of her child, the arousal of emotional states and can repair any child emotional dysregulation [88]. This transition from a mismatched or negative state into a matched or positive state, referred to as ‘repair’ [89], is the process by which children internalise regulatory abilities [90, 91]. Another important aspect of early shared reading is that it can be considered a ‘transitional space’ [92], in which the child experiences his own fantasies, expectations and hypotheses, and shares representations of reality with others. In a suitable shared reading context, the parent becomes more available and sensitive to the child, dedicates attention and time to them in a ‘potential space’ in which the parent presents the book (object presenting) in a playful and creative way.

The DBS intervention involves cognitive, socio-emotional and affect-regulating elements, as well as well-being in the parent–child relationship, and we believe that this psycho-educational intervention is also important as a support for parenting. These positive emotional effects of moments of interaction in a privileged intersubjective space are also extendable to the parent. The possibility of being involved in pleasant activities with the child could also have positive effects in cases of parental affective disorders (depression, anxiety, stress). The parent can be pleasantly involved with the child, and the book can be used in the interaction as a resource when the relationship may otherwise present communication challenges for the parent. In parent–child interaction, the parent’s emotional state is of fundamental importance, and a parent with affective disorders, oriented towards his or her inner problems [93, 94], may have greater difficulties in relating intersubjectively with their child, with long-term effects on the child’s cognitive development at later ages [95]. The illustrated book with colourful and interesting images, therefore, can be considered as a ‘mediator’ between adult and child, as it could activate a positive emotional state in the parent and involve them more in interaction with the child.

We propose conducting a trial of DBS in a study ‘Early parent–child intervention with Dialogic Book-Sharing: effects on child communicative and socio-emotional development and on parenting’. Not only could the DBS programme promote benefits to child development and parenting, but providing parenting training in a small group could also represent a moment for the promotion of sociality among families, especially relevant as a response to the social, health and educational needs of the post-COVID-19 pandemic era caused by the social isolation measures adopted to curb the spread of the virus. Finally, it is a way to promote the use of books and counteract the excessive use of technological devices already present in early childhood.

### Objectives

The objectives of the study are to evaluate the impact of a Dialogic Book-Sharing programme delivered to parents within 12 health, educational and maternal-child centres, on (a) the child’s linguistic, cognitive, attention and behavioural development; (b) the parent–child interaction; (c) the parent’s mood (anxiety, depression and stress) and (d) decreasing amount of screen time compared to an increase in parent–child book sharing at home.

## Methods

### Selection of health, educational and maternal-child centres

The Observatory of Perinatal Clinical Psychology at the University of Brescia has invited health, educational and maternal-child centres that indicated an interest in taking part in a study of the impact of a dialogic book-sharing training provided to parents. Following approval from each centre’s director, 12 centres were formally accepted into the study (see Table 1). In each participating centre, local staff were identified to run the research project within their centre, with minor adaptations to accommodate their own operational reality. These local staff are responsible for over-seeing identification and recruitment of families, baseline data collection, intervention delivery and follow-up assessment of study participants.

**Table 1:** Health, educational and maternal-child centres

Location	Unit type	Professionals involved
Brescia	Nursery school	Pedagogist
Brescia	Mother–child protected community	Psychologist Professional educator
Bergamo	Nursery school	Pedagogist
Milan	Nursery school	Pedagogist
Mantua	Nursery school of Carlo Poma Hospital	Psychologist
Mantua	Nursery school	Psychologist
Como	Nursery school	Pedagogist
La Spezia	Family centre	Psychologists
Florence	Nursery schools	Psychologist
Rome	Nursery schools of XIV Municipio Comune di Roma	Psychologist
Naples	Nursery school	Psychologist
Salerno	Gynaecology and obstetrics unit/nursery/PMA centre	Psychologist

During the study, there is regular contact between the coordinating centre (the Observatory of Perinatal Clinical Psychology) and the participating health, educational and maternal-child centres. Weekly conference calls are held, as are telephone consultations and periodic meetings to monitor the study progress and to keep all coordinators abreast of the progress of the project.

### Training programme for healthcare professionals

Several meetings with the participating health, educational and maternal-child centres have taken place to plan and finalise the project organisation. Profs Lynne Murray and Peter Cooper of the University of Reading (UK) have adapted the Dialogic Book-Sharing programme that they developed and have successfully previously trialled in South Africa and the UK [17, 48–50, 60], specifically for the current project. The core feature is that parents are trained in how to support their child’s interest and active engagement, rather than simply ‘reading’ to their child. Responsiveness is emphasised that is sensitive to the child’s developmental capacity and experience, as well as the importance of a positive encouraging approach. The intervention is being delivered in centres by local staff who, over a 2-day training course, have been trained (by Dr Mauro Belluardo of University of Parma) to act as facilitators. Participants receive ongoing supervision from LM and PJC. In accordance with the literature (e.g. [96]), the supervision sessions help to ensure fidelity with programme delivery (see details below in ‘Measures’). The intervention programme is delivered within each centre in Italian, using PowerPoints and embedded demonstration videos. The course is structured into four, weekly, meetings of the parents lasting 90 min each, which take place at the health, educational and maternal-child centres.

The sessions are conducted with groups of up to six parents, during which children are cared for in an adjoining play space by centre staff. In each session, the facilitator presents parents with different ways to share the book with their children, according to a defined programme. At the beginning of the training course, the trained facilitator promotes discussion among the parents and the sharing of their personal experiences within the group in order to facilitate a collective commitment to the programme. This sense of corporate endeavour is maintained throughout the programme by group discussion of progress. At the end of each group session there is a brief period of one-to-one interaction between the facilitator and each parent together with their child, who are given a book to take home with them, and they are given support and encouragement in applying the acquired dialogic book-sharing techniques with their child using this book. After being exposed to new techniques of book-sharing during each group session, parents are advised to apply these techniques autonomously during the following week in their daily life with their children. After the four sessions of the intervention, parents will be encouraged to continue to apply the full set of techniques with their child over the next 6 months.

### Recruitment

**Fig. 1:**
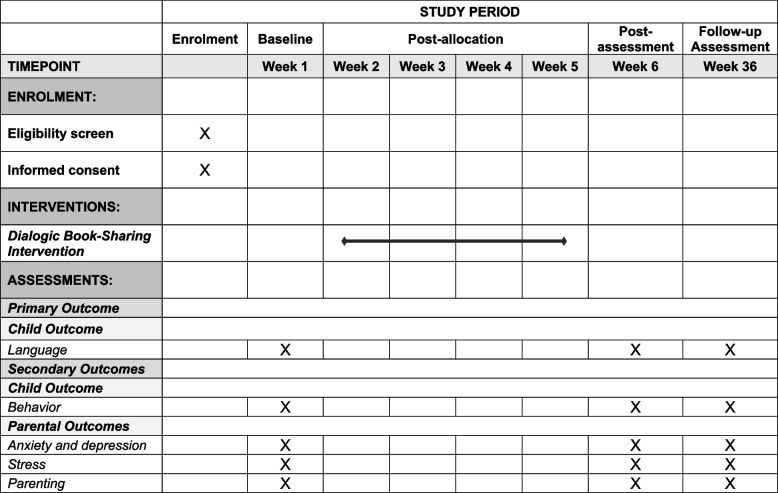
Schedule of enrolment, interventions and assessments

All parent–child dyads attending the included health, educational and maternal-child centres who meet the inclusion criteria (see below) are being invited by local staff to participate in the study. Participation is voluntary and no charge is levied for receiving the training course. Those parents who agree to participate sign the consent form in which it was made clear that they could withdraw from the study at any time without explanation (Fig. 1).

### Selection criteria

#### Inclusion criteria

- Mothers and fathers who are able to speak and read Italian
- Children aged 14–20 months at the time of the baseline assessment

#### Exclusion criteria

- Children who have a diagnosed physical or intellectual impairment
- Mothers and/or fathers with a diagnosed physical or psychiatric condition that could compromise their ability to participate in the intervention programme

### Study design

This is a multicentre, two parallel arms, single blinded randomised controlled superiority trial, with a 1:1 arms allocation ratio. The study followed the SPIRIT reporting guidelines (Supplementary File).

Within each health, educational and maternal-child centres, parent–child dyads are randomly assigned by local staff to either the index group or the control condition based on a randomisation list compiled using random blocks algorithm. The index group receive the DBS intervention immediately, and the control group enter a waiting list and receive the DBS intervention after the final study assessment. The local staff, not involved in the participant treatment and blinded to treatment allocation, collect the baseline data during an interview before randomisation and again after the four group sessions. A follow-up evaluation is to be carried out 6 months after the end of the training.

### Sample size estimation

The sample size was calculated based on the difference of the number of words between the two study groups (index and control group) at post-test. In the Dowdall meta-analysis, the average effect size for expressive language adjusted for baseline pre-test value was 0.41. Taking this value as the reference to compute the sample size for our study, assuming a *t*-test for independent samples, a two-sided 5% significance level, a sample size of 190 (95 in each groups) will provide a power of at least 80% (PASS 2021).

### Measures

In each assessment session, the parent is invited to participate in a video recording of a 5-min parent–child interaction and asked to complete a self-report questionnaire taking approximately 30 min.

#### Socio-demographic information

We are obtaining information on the parent’s socio-demographic details (age, nationality, educational level, professional occupation, economic condition) and information about pregnancy and post-partum period.

### Outcomes

The primary outcome is the child’s language development. Secondary outcomes are the child’s behaviour, parental mood (anxiety, depression and stress) and parenting.

### Primary outcome

#### The MacArthur-Bates Communicative Development Inventory (CDI)

Parents are asked to complete the short form of the CDI [97], one of the most widely used and recommended measures of language and communication for young children. This provides an assessment of the expressive and receptive vocabulary of 8–36-month infants. Parents identify words that the child is able to understand and those that the child can enunciate from a 100-word checklist. A raw score for expressive and for receptive language is obtained. The Italian version of the CDI [98] has been employed in several cross-cultural studies investigating the linguistic development of young children [99, 100]. The instrument showed adequate validity: high correlations between parent-report scores and child performance on concurrent standardised tests have been reported [101–103].

### Secondary outcomes

i) Child outcome.

#### Child behaviour

The Achenbach System of Empirically Based Assessment (ASEBA) is being used to assess child behaviour [104] with the Child Behavior Checklist 1½-5 (CBCL), a widely used parent report checklist that measures a broad range of behavioural and emotional problems among young children. Parents complete the questionnaire by providing ratings to descriptive statements (0 = not true, 1 = somewhat or sometimes true, 2 = very true or often true). For the current study, the questionnaire comprises 27 items that provide an index of Emotionally Reactive, Attention Problems, Affective Problems and Attention Deficit/Hyperactivity Problems. The CBCL’s scales demonstrated good instrument quality and validity and showed good psychometric properties with regard to consistency, reliability and cross-informant agreement [105].

ii) Parental outcomes.

#### Anxiety and depression

To assess parental mood, the Hospital Anxiety and Depression Scale (HADS) is being used [106]. This is a 14-item self-report questionnaire which aims to identify the presence of anxious and depressive symptoms. Each item is rated on a 4-point Likert scale reflecting severity. The HADS is completed on the basis of the emotional state felt over the previous week. Although it was initially developed for patients in hospital settings, several studies have shown that it is appropriate in a wide range of setting, including within the general population [107]. The validation of the original version of this psychometric scale was followed by translations into various languages, including Italian [108].

#### Parenting stress

The short form of the Parenting Stress Index (PSI-SF) [109] is being used. This measure is commonly used to assess parenting stress both in clinical and research contexts. For the current study, we only administer one subscale of the PSI-SF, namely Parent–Child Dysfunctional Interaction (P-CDI). This consists of 12 items rated from 1 (strongly disagree) to 5 (strongly agree) assessing the extent to which parents feel satisfied with their child and their interactions with them.

#### Parenting

The secondary outcome is parenting which is assessed on all three data collection occasions by direct observation. Parents are asked to share the same text-light book with their child (‘Yes’ by Jez Alborourgh) in the way they would at home. The interactions are filmed. Five minutes of interaction are rated on measures of parental behaviour and parent–child interaction [48]. The measures concern dimensions of book-sharing that the intervention is designed to enhance, and they therefore provide an objective measure of how well parents had implemented the strategies covered in the training programme. Videos and transcripts are scored by trained researchers blind to group and child outcome. Random samples will be scored by independent trained assessors to establish interrater reliability. The principal dimension of parenting assessed is sensitivity*.* This concerns parental appropriate and warm responsiveness to the child [48, 49]. Key aspects include the parent’s awareness of the child’s focus of interest (e.g. gaze direction, pointing, efforts to turn the page) and their communication, as well as the extent to which parental responses to these behaviours were supportive and well timed. The level of sensitivity is rated from the videos on a 5-point scale.

Reciprocity is also rated from these videos. This concerns shared affect (e.g. smiles, expressions of surprise, concern) and joint attention to the book (e.g. gazing and pointing together at the same part of the page), vocal exchanges and gestural turn taking (e.g. stroking motions on depicted book characters) and mutual gaze [48, 49], and was rated from the videos (score = rating on 5-point scale).

### Statistical analysis plan

Descriptive statistics will be performed on socio-demographic and baseline child cognitive-behavioural variables using summary statistics such as mean, standard deviation, median and interquartile range. Comparison of test scores measure, derived as combination of individual score items, at post-test between the two groups will be performed using a linear mixed model accounting for baseline (pre-test) test scores values and a random term to account for recruitment centre effect. Individual items measured on an ordinal scale will be compared using cumulative link models for ordinal measures. Results will be reported as effect estimates and corresponding 95% confidence intervals. All tests will be two-sided and will be evaluated assuming a 5% significance level. This analysis will be performed by an independent statistician, blinded for the treatment allocation. The statistician will report to the Data Monitoring Committee. Data will be analysed based on intention to treat principles. A sensitivity analyses will also be performed, excluding not-adherent participants from the analyses.

## Discussion

The Dialogic Book-Sharing programme will be implemented in Italian health, educational and maternal-child centres through the study ‘Early parent–child intervention with Dialogic Book-Sharing: effects on child communicative and socio-emotional development and on parenting’. It will represent an early parent–child intervention in the first thousand days of life and will aim to explore the impact of the DBS method on the child’s linguistic, cognitive, attention and behavioural development in the first 2 years of life, on parent mood (anxiety, depression and stress) and parent–child interactions.

The implementation of the DBS psycho-educational programme in early childhood could enhance the educational resources offered by educational, social and health services, early childhood centres and maternal-child services, in support of parenting and the parent–child relationship. This intervention could represent added value for assistance to families, in terms of the improvement of the psychophysical well-being, the quality of life of the child and their parents, as well as the social well-being of the territory.

The provision of the DBS model has been found to be feasible, acceptable and effective in different ecological contexts and educational services [48, 60, 61, 110]. It represents a strategic opportunity for bringing about positive effects, also in terms of prevention of socio-emotional and cognitive difficulties, and for the promotion of the psychosocial health of the population that attends Italian health, educational and maternal-child centres. As such it represents a promising response to the new social, health and educational needs currently accentuated by the COVID-19 pandemic and the imposition of social isolation adopted by governments to curb the spread of the virus, with consequent adverse impact on the most vulnerable populations [111, 112].

Furthermore, the application of the DBS methodology is also a way to promote the use of books, and thereby counteract the excessive use of technological devices already present in early childhood. In the past decade, screen time has become ubiquitous in children’s daily routines, with an impact across multiple domains of their development [113]: an excessive exposure to screen resulted negatively associated with developmental health outcomes in children [114] since it might harm their cognitive [115], linguistic and social-emotional growth [116].

### Supplementary Information

Additional file 1. SPIRIT checklist.

## Data Availability

The data that support the findings of this study are available from the corresponding author upon reasonable request.

## Abbreviations

ANOVA: Analysis of variance
ANCOVA: Analysis of covariance
ASEBA: Achenbach System of Empirically Based Assessment
CBCL: Child Behavior Checklist
CDI: Communication Development Inventory
COVID-19: Coronavirus disease 2019
DBS: Dialogic Book-Sharing
HADS: Hospital Anxiety and Depression Scale
MNs: Mirror Neuron System
P-CDI: Parent-Child Dysfunctional Interaction
PSI-SF: Parenting Stress Index
RCT: Randomised controlled trial
